# Characterization of temporal electrical activity patterns for detection of critical isthmus regions of recurrent atypical atrial flutter

**DOI:** 10.1002/clc.24009

**Published:** 2023-03-27

**Authors:** Nadine Vonderlin, Johannes Siebermair, Amir Mahabadi, Elena Pesch, Miriam Koehler, Dobromir Dobrev, Rolf Alexander Janosi, Tienush Rassaf, Reza Wakili

**Affiliations:** ^1^ Department of Cardiology and Vascular Medicine, West‐German Heart and Vascular Center Essen, University of Essen Medical School University Duisburg‐Essen Essen Germany; ^2^ German Centre for Cardiovascular Research (DZHK) Berlin Germany; ^3^ Institute of Pharmacology, West German Heart and Vascular Center University Duisburg‐Essen Essen Germany; ^4^ Department of Molecular Physiology and Biophysics Baylor College of Medicine Houston Texas USA; ^5^ Department of Medicine and Research Center, Montreal Heart Institute Université de Montréal Montréal Quebec Canada; ^6^ Department of Cardiology and Vascular Medicine, University Hospital Frankfurt Goethe University Frankfurt Germany

**Keywords:** atypical atrial flutter, isthmus, Lumipoint®, re‐entry

## Abstract

**Introduction:**

Identifying the critical isthmus region (CIR) of atrial re‐entry tachycardias (AT) is challenging. The Lumipoint® (LP) software, developed for the Rhythmia® mapping system, aims to facilitate the successful ablation of ATs by identifying the CIR.

**Objective:**

The objective of this study was to evaluate the quality of LP regarding the percentage of arrhythmia‐relevant CIR in patients with atypical atrial flutter (AAF).

**Methods:**

In this retrospective study, we analyzed 57 AAF forms. Electrical activity (EA) was mapped over tachycardia cycle length resulting in a two‐dimensional EA pattern. The hypothesis was that EA minima suggest potential CIRs with slow‐conduction‐zone.

**Results:**

A total of *n* = 33 patients were included, with the majority of patients being already preablated (69.7%). LP algorithm identified a mean of 2.4 EA minima and 4.4 suggested CIRs per AAF form. Overall, we observed a low probability of identifying only the relevant CIR (POR) at 12.3% but a high probability that at least one CIR is detected (PALO) at 98.2%. Detailed analysis revealed EA minima depth (≤20%) and width (>50 ms) as the best predictors of relevant CIRs. Wide minima occurred rarely (17.5%), while low minima were more frequently present (75.4%). Minima depth of EA ≤ 20% showed the best PALO/POR overall (95% and 60%, respectively). Analysis in recurrent AAF ablations (five patients) revealed that CIR in de novo AAF was already detected by LP during the index procedure.

**Conclusion:**

The LP algorithm provides an excellent PALO (98.2%), but poor POR (12.3%) to detect the CIR in AAF. POR improved by preselection of the lowest and widest EA minima. In addition, there might be the role of initial bystander CIRs becoming relevant for future AAFs.

## Graphical abstract

In an unselected cohort of 33 patients presenting with *n* = 57 atypical atrial flutter forms application of the Lumipoint (LP) algorithm with preselection by electrical minima of ≤20% resulted in a probability of >90% of identifying a critical isthmus region (CIR) and probability of 60% of specific CIR only identification.

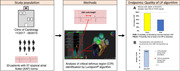

## 1 INTRODUCTION

Atrial scar regions favor the formation of slow‐conducting areas in the myocardial chambers and thereby contribute to the occurrence of re‐entry tachycardias, for example, atypical atrial flutter (AAF). This is frequently observed in preablated patients, especially after pulmonary vein isolation (PVI). Studies show that over 30% of patients develop clinically relevant atrial tachycardia (AT) during follow‐up (FU).^1^ Ablation of those ATs is often difficult due to heterogeneity of the underlying substrates, variability of cycle length (CL), varying forms of atrial flutter, and different locations in the atrium.^2^, ^3^, ^4^, ^5^ Essential for the successful treatment of re‐entry tachycardia is the identification of the underlying mechanism. The critical isthmus region (CIR) usually corresponds to a slow conduction zone building the pathophysiological basis for electrical re‐entry.^6^ Because of slow conduction in a localized area of cardiac tissue, a low cumulative electrical activity (EA) of cardiomyocytes over time is resulting in low amplitude intracardiac electrograms (EGMs). Hence, regions with low EA over time are suspected to represent slow conduction areas due to diseased tissue or remodeling, for example, fibrosis. Translating this hypothesis to a surface electrocardiogram (ECG) isoelectric phases of the ECG between p‐waves are suspected to represent the time of electrical slow conduction through the CIR.^7^ Since these CIR are crucial for tachycardia initiation and maintenance, the identification and ablation of the CIR is the aim of electrophysiological studies targeting re‐entry tachycardias.

Lumipoint® (LP) is a novel software algorithm, developed for the Rhythmia® mapping software, which aims to identify the CIR of re‐entrant tachycardias by applying the theory of low EA representing potential CIRs.^8^, ^9^ The feature detects the temporal distribution of the mapped electrical activation by analysis of all single EGM to determine activity at each location depending on local activation time (LAT).

**Figure 1:**
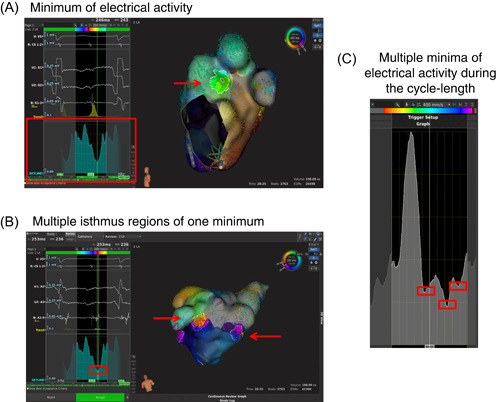
(A) Left atria, view from the left. “Skyline” tool displays on the right the histogram of global atrial surface activation during AAF cycle length, which shows the relative proportion of the atrial surface area that is activated at each point in time (electrical activity *X* axis, cycle length *Y* axis). The green bar (length of 30 ms) marks the minimum (red box) of the skyline, which shows one isthmus (highlighted region, red arrow) in the dorsal wall of LA. (B) Left atria, view from dorsal. One minimum involves two highlighted regions (red arrows) as potential CIR. (C) Example of three minima in the “skyline.” AAF, atypical atrial flutter; CIR, critical isthmus region; LA, left atria.

The tool “skyline” generates a two‐dimensional (2D) histogram in which a full chamber activation is displayed (see Figure 1A, red box). It can be used to highlight regions of interest on the map that activate within a certain time of the cycle.^8^ Peaks in the histogram correspond to areas with high cumulative EA while a valley is seen as having very low EA surface activation. The latter is the point of interest cause areas of minimal activation may correspond to slow conduction zones and therefore to the potential CIR of the AAF.

To evaluate if the new algorithm LP improves the interpretation of complex atrial macro re‐entry tachycardia and identify potential ablation strategies, we performed this retrospective study. The objective was to determine the quality of the algorithm for determining the CIR of re‐entrant AAF tachycardias. Furthermore, we sought to investigate if LP can predict relevant CIR for future clinically relevant ATs.

## 2 METHODS

### 2.1 Study population

In this is a retrospective, single‐center study; in total *n* = 33 patients were included, who underwent ablation by ultrahigh‐density mapping guided AT ablation with the Rhythmia® mapping system in our center from November 2017 to September 2019. The mean FU time was 10 ± 7 months. All patients gave written informed consent before ablation. The methods were carried out in accordance with the relevant guidelines and regulations of the University of Duisburg‐Essen. The protocol was approved by the institutional review board and the local ethics committee (registration number: 19‐8714‐BO).

### 2.2 Electrophysiological study and mapping

Antiarrhythmic drugs (AADs), if administered, were continued during the study period. All patients were on oral anticoagulation therapy at least >24 hours before the catheter ablation date. Atrial thrombus was excluded by transesophageal echocardiography in every patient <3 days before the procedure. Throughout the procedure, an activated clotting time of >300 seconds was aimed for.

During the procedure, a steerable decapolar catheter was placed in the coronary sinus (CS).

We used the Orion™ multipolar basket catheter and Rhythmia™ system (both Boston Scientific) for the mapping of AAF. A local activation (LAT) map was generated with an automatic standard beat acceptance criteria based on the annotations algorithm: (1) CL variation, (2) activation time difference variations between the CS EGMs, (3) propagation reference (ΔR), (4) respiration, (5) QRS morphology “favorite beat” (ECG), (6) mapping catheter movement, (7) EGM stability compared to last beat, (8) tracking quality, and (9) window. Entrainment mapping was not performed.

### 2.3 Ablation approach

Based on the atrial LAT map, the most likely dominant AAF mechanism and identification of the circuit were determined. Appropriate ablation sites were selected in dependence on the anatomic distance, catheter stability, tissue thickness, and vulnerable structures located nearby. Mostly, the selected ablation side was a narrow scope between scars and anatomical obstacles. In theory, several ablation sites can lead to the termination of the AAF, practical ablation site was chosen at the discretion of the operator. Independent of the selected isthmus, the aim of ablation was an ablation line resulting in a bidirectional block.^10^, ^11^, ^12^ PVI was obtained in the same procedure if needed. We used a 3.5 mm tip ablation catheter (Thermocool®; Biosense Surround Flow; Biosense Webster) to generate radiofrequency energy with a maximal 35 W (30 W at the posterior wall). Each RF ablation was limited to 240 seconds, delivered by a 500 kHz ablation unit (Stockert EP shuttle; Biosense Webster Inc.).

### 2.4 LP algorithm

In our study, we applied the LP algorithm retrospectively. LP offers the possibility to apply three different tools “skyline,” fractioned ECGs, and double potentials. In our study, we focused on the first tool “skyline” which creates a 2D map of EA over the CL of the tachycardia. Thereby, the tool uses the information of full chamber surface activation. The result is the “skyline” which plots the relative proportion of the atrial surface area that is activated in time throughout the entire CL (Figure 1A). The result is a normalized global activation histogram which values ranging from 0.01 to 1.0 (for our calculations 1%–100%). “Peaks” correspond with points of maximal electrical activation when a large part of the atrium is being activated and were called maximum in our analyses. The points of interest are the “valleys” or “minima” of EA (Figure 1B), which represent potentially the CIR as a surrogate of slow conduction.^9^, ^13^

In our study, we analyzed the “skyline” pattern as follows: a minimum was accepted if the selected EA's relative value was <50%, and peaks before and after had to be at least ≥5% higher than the minimum of interest. Further additional minima were included if the EA value did not exceed the lowest minimal EA point more than twofold (Figure 1C).

By starting the “activation search” feature of LP, a predefined unit time of 30 ms (green bar in Figure 1A) scans the electrical activation map. At every moment, the local activated areas were shown at the atrium surface. When the green bar reaches a predefined potential “isthmus” regions with minimal cumulative EA over the CL of the tachycardia were highlighted. A minimum could contain several highlighted areas and therefore several isthmi (e.g., see Figure 1B: 1 minimum with 2 isthmi). The definition of CIR was based on the assumption that ablation would lead to the termination of AAF, always re‐evaluated by a successful termination under ablation confirming the correct initial suspected re‐entry mechanism.

Further, we analyzed the width and depth of EA minima in detail and categorized them into five groups with respect to depth (0%–10%, >10%–20%, >20%–30%, >30%–40%, and >40%–50% of EA) and seven groups according to width (0–10 ms, >10–20 ns, >20–30 ms, >30–40 ms, >40–50 ms, >50–100 ms, and >100–160 ms).

### 2.5 Study endpoints

The primary study endpoint was to evaluate the quality of the selection of proposed isthmi by the LP algorithm. We aimed for determining the probability of identifying only the relevant CIR (POR) and the probability that at least one CIR is detected (PALO) as well as possibilities to improve the algorithm by further characterization of minima.

Secondary endpoints comprised the analysis of the total number of the detected isthmus, as well as their number per AFF and minimum. Furthermore, we evaluated the recurrence rate of AAF during FU, the number of patients who underwent reablation, and if potential LP‐suggested CIR regions during index procedure were involved in future recurrent clinical ATs in the individual patient.

### 2.6 Statistical analysis

Data in our study are expressed as mean (±standard deviation) for continuous variables or as numbers and percentages for categorical variables. For categorical variables, *χ* ^2^ analysis or Fisher's exact test was applied. Statistically, significance was considered by a *p* < 0.05.

## 3 RESULTS

The analysis of the included 33 patients revealed a mean number of 1.7 AAF forms per patient. In total, we analyzed 57 atrial macro re‐entry tachycardia forms. Out of these, *n* = 49 AAF were localized in the left atrium and *n* = 8 in the right atrium, but not involving the tricuspid isthmus. In all cases, targeted ablation of AAF leads to successful termination or conversion into another AAF form.

### 3.1 Patient characteristics

The baseline characteristics of the 33 patients are listed in Table 1. The mean age was 70.6 years, and more than half of the patients were male (54.5%). An echocardiographic examination revealed a preserved ejection fraction of the left ventricle. Most of the patients (90.9%) were treated with at least one AAD; in most cases, beta‐blockers (90.9%), followed by amiodarone and flecainide (24.2% and 9.1%, respectively). The most common cardiovascular risk factor was arterial hypertension (84.8%). Almost 9% suffered from a stroke or TIA in the past and 45.5% had a diagnosis of coronary artery disease. In addition, 45.5% of patients were equipped with a cardiac device (pacemaker, implantable cardiac defibrillator, or cardiac resynchronization therapy‐defibrillator/pacemaker). Most patients (84.8%) had a previous history of cardiac intervention. In most cases, patients were preablated in past (69.7%), of which 51.5% did undergo a PVI before. A history of prior cardiac surgery was the case in 24.2%, and of a transcatheter mitral valve repair or valvuloplasty in 9.1%.

**Table 1:** Patient characteristics; *n* = 33.

Baseline characteristics	Overall (*n* = 33)
Age (years)	70.6 ± 13.2
Male, *n* (%)	18 (54.5)
Previous cardiac procedures, *n* (%)	28 (84.8)
Ablation, *n* (%)	23 (69.7)
PVI, *n* (%)	17 (51.5)
Postcardiovascular surgery, *n* (%)	8 (24.2)
Postinterventional procedure, *n* (%)	3 (9.1)
LVEF, *n* (%)	50.2 ± 10.0
Medication (AAD), *n* (%)	30 (90.9)
Beta‐blocker, *n* (%)	30 (90.9)
Amiodaron, *n* (%)	8 (24.2)
Flecainide, *n* (%)	3 (9.1)
TIA/stroke in history, *n* (%)	3 (9.1)
Coronary artery disease, *n* (%)	15 (45.5)
Arterial hypertension, *n* (%)	28 (84.8)
Diabetes mellitus, *n* (%)	6 (18.2)
Chronic kidney disease (GFR < 60 mL/min/1.73 m^2^), *n* (%)	19 (57.6)
GFR > 30 mL/min/1.73 m^2^, *n* (%)	18 (54.5)
GFR < 30 mL/min/1.73 m^2^, *n* (%)	1 (3.0)
Device, *n* (%)	15 (45.5)
Pacemaker, *n* (%)	11 (33.3)
1 chamber ICD, *n* (%)	2 (6.1)
CRT‐D, *n* (%)	2 (6.1)

*Note*: Data are presented as *n* (%) or mean and standard deviation. The mean age was 70.6 years, more than half of the patients were male and most of the included patients took an AAD.

Abbreviations: AAD, antiarrhythmic drugs; CRT‐D, cardiac resynchronization therapy‐defibrillator; GFR, glomerular filtration rate; ICD, implantable cardiac defibrillator; LVEF, left ventricular ejection fraction; PVI, pulmonary vein isolation; TIA, transitory ischemic attack.

### 3.2 Electrophysiological characteristics

Table 2 shows the electrophysiological characteristics and results of the skyline analysis of the mapped AT. Overall, 57 AAFs (1.7 AAF per patient) were analyzed in this retrospective study. The mean CL of the AAFs was 291.0 ± 70.2 ms. The mean total mapping time was 12.1 ± 7.0 minutes. In total, 56/57 (98.2%) AAFs were terminated by ablation, while only one AAF (1.8%) converted to atrial fibrillation (AFib) under ablation.

**Table 2:** Electrophysiological characteristics; the number of atrial tachycardia *n* = 57.

Electrophysiological characteristics	
Number of AAFs, *n*	57
AAFs in LA, *n* (%)	49 (86.0)
AAFs in RA, *n* (%)	8 (14.0)
Number of AAFs/patient, *n*	1.73
Cycle length of AAF, ms	291.0 ± 70.2
Mapping time of atria, min	12.1 ± 7.0
Conversion in sinus rhythm by ablation, *n* (%)	56 (98.2)
Number of minima, *n*	137
Number of minima/AAF, *n*	2.4
Number of isthmi, *n*	248
Number of isthmi/AAF, *n*	4.4
Number of isthmi/minimum, *n*	1.8
Minima characteristics	
Level of EA minima, %	22.7 ± 13.0
Width of minima, ms	24.5 ± 22.1
EA peak preminimum, %	65.2 ± 28.4
EA peak postminimum, %	64.0 ± 27.3

*Note*: Data are presented as *n* (%) or mean and standard deviation. On average the cycle length was 291 ms and the mapping time of the involved AAF was 12.1 minutes. The mean number of minima per AAFs was 2.4, and the mean number of isthmi was 4.4.

Abbreviations: AAF, atypical atrial flutter; EA, electrical activity; LA, left atria; RA, right atria.

The skyline analysis revealed a total number of 137 minima, the mean number of minima per AAF was 2.4. Supporting Information: Figure 1A depicts the detailed numbers and distribution of minima per AAF (1 minimum/AAF: 25%; 2 minima/AAF: 26%; 3 minima/AAF: 39%; 4 minima/AAF: 5%; 5 minima/AAF: 5%). The total number of highlighted areas representing the number of potential isthmi was *n* = 248. LP identified 1.8 isthmi per minimum and 4.4 isthmi per AAF in the whole cohort. Supporting Information: Figure 1B shows the distribution of isthmus number per minimum (1 isthmus/minimum: 46%; 2 isthmi/minimum: 32%; 3 isthmi/minimum: 17%; 4 isthmi/minimum: 5%). In addition, Supporting Information: Figure 1C illustrates the number of isthmi per AAF (1 isthmus/AAF: 12%; 2 isthmi/AAF: 18%; 3: isthmi/AAF: 12%; 4 isthmi/AAF: 16%; 5 isthmi/AAF: 16%, ≥6 isthmi/AAF: 26%).

### 3.3 Quality of CIR detection

**Figure 2:**
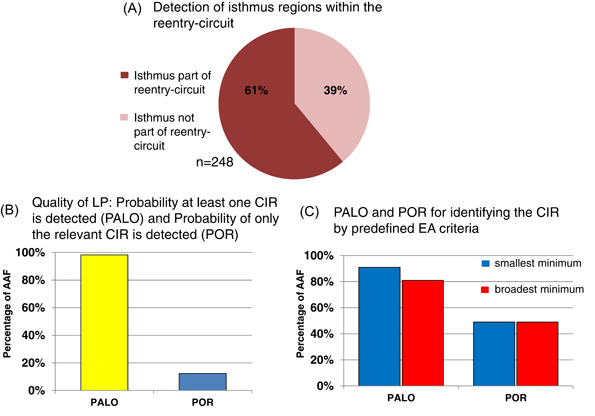
(A) Proportion of detected isthmi, which are part of the AAF re‐entry circuit (61%). (B) PALO and POR of the LP algorithm with respect to the detection of the AAF‐relevant CIR. PALO represents the detection of at least one relevant CIR by LP, which was 98.2%. POR was defined as the detection of only re‐entry‐relevant CIR, which was 12.3% in the unselected all‐over analysis. (C) PALO and POR after applying the preselection criteria: analyzing the smallest electrical activation minimum only (left); selecting the broadest electrical activation minima (right). AAF, atypical atrial flutter; CIR, critical isthmus region; EA, electrical activity; LP, Lumipoint; PALO, probability of identifying at least one CIR; POR, probability of identifying only the relevant CIR.

In the next step, we evaluated the potential involvement of the LP‐suggested isthmi in the re‐entry circuit and the opportunity for AAF termination at this region by ablation. Our analysis revealed that 60.9% (151/248) of suggested isthmi were part of the re‐entry as shown in Figure 2A. Analysing the PALO and the POR of the LP algorithm showed a high PALO of 98.2% (56/57) but a very low POR of 12.3% (7/57) (Figure 2B). To further improve POR, we analyzed different characteristics of the minima and evaluated if or how applying distinct preselection criteria would translate into higher POR. The mean width of a minimum was 24.5 ± 22 ms, while the mean EA level was obtained at 22.7 ± 13.0%, always related to the maximum level (100%) of the EA in the skyline pattern. The mean peak preceding as well as after a minimum were mostly located between 60% and 65% of maximum EA (65.2 ± 28.4% and 63.7 ± 27.5%, respectively).

Our analysis revealed that in case we select the widest minimum only (mean 35.9 ± 29.4 ms), we had a decrease in PALO down to 80.7% (46/57; CIR was involved in longest minima) but an improved POR of 49.1% (28/57). The best result was yield when the lowest/deepest minimum (average 14.4 ± 9.8%) out of all suggested was chosen. In this setting, the PALO was still high at 91.2% (Figure 2C) and the POR increased up to 49.1% (28/57) when compared to the baseline condition.

In addition, we further characterized the minima regarding its width with respect to predicting the potential CIR. Supporting Information: Figure 2A shows, deeper minima are associated with an improved POR as well as PALO in comparison to higher minima (0%–10%: PALO 91%, POR 56%; >10%–20%: PALO 80%, POR 49%; >20‐30%: PALO 44%, POR 13%; >30%–40%: PALO 50%, POR 9%; >40%–50%: PALO 44%, POR 0%). With regard to the length (Supporting Information: Figure 2B), longer minima showed better PALO as well as POR in contrast to shorter minima (0–10 ms: PALO 74%, POR 16%; >10–20 ms: PALO 51%, POR 22%; >20–30 ms: PALO 66%, POR 34%; >30–40 ms: PALO 92%, POR 33%; >40–50 ms: PALO 67%, POR 33%; 50–100 ms: PALO 100%, POR 75%; >100–160 ms: PALO 100%, POR 100%). In the next step, we analyzed the deepest minima of each AAF (*n* = 57); here we also found an improved PALO and POR of deeper minima in comparison to higher minima (see Supporting Information: Figure 2C; 0%–10%: PALO 93%, POR 62%; >10%–20%: PALO 100%, POR 57%; >20%–30%: PALO 80%, POR 20%; >30%–40%: PALO 67%, POR 0%; >40%–50%: PALO 100%, POR 0%). Taken together, the lowest minima characterized by a depth of EA ≤ 20% (43/57 cases), showed a very good PALO as well as POL (95% and 60%, respectively).

Finally, we compared the longest minima of each AAF (see Supporting Information: Figure 2D). Wide minima were associated with an improved prediction of potential CIR (0–10 ms: PALO 100%, POR 0%; >10–20 ms: PALO 50%, POR 21%; >20–30 ms: PALO 89%, POR 44%; >30–40 ms: PALO 89%, POR 44%; >40–50 ms: PALO 67%, POR 33%; 50–100  ms: PALO 100%, POR 86%; >100–160 ms: PALO 100%, POR 100%). In conclusion, minima longer than 50 ms (in 10/57 cases), had an excellent PALO with 100% as well as POR with 90%.

In our study, eight patients (24.2%) underwent cardiac surgery (postop) in the past and developed 14 AAF forms. For a better understanding, we compared PALO and POR between this specific postop subgroup and the overall AAF group revealing no relevant difference for PALO and POR between the groups (PALO 98.2% overall vs. 100% postop, *p* = 1.0; POR 12.3% overall and 7.1% postop, *p* = 1.0; see also Supporting Information: Figure 3A). Regarding the criterion of “only smallest minimum,” we observed a small, but not significant difference between both groups in favor of the postop AAF forms (PALO 91.2% overall vs. 100% postop, *p* = 0.57; POR 49.1% overall vs. 71.4% postop, *p* = 0.15; Supporting Information: Figure 3B, online). When analyzing the smallest minimum characterized by EA ≤ 20%, again PALO and POR were slightly better in postop AAF (applicable in 13/14 cases) compared to overall AAF forms (applicable in 43/57 cases) (PALO 91.2% overall vs. 100% postop, *p* = 1.0; POR 60.5% overall vs. 76.9% postop, *p* = 0.34; Supporting Information: Figure 3C).

**Figure 3:**
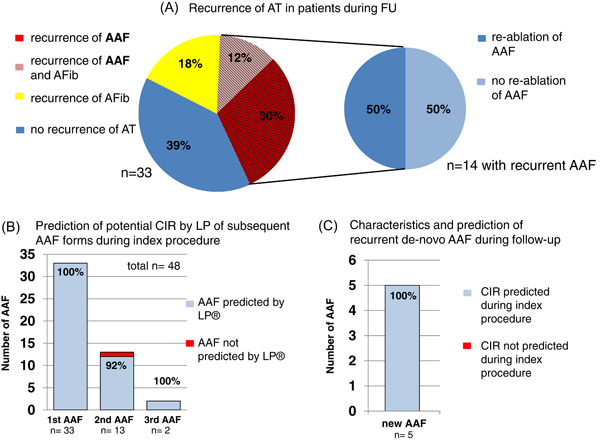
(A) Clinical outcome: Recurrence of AT in the FU of 10 ± 7 months. 20/33 patients have another AT: 30.3% of patients have another AAF, 18.2% have a recurrence of AFib, and 12.1% cases both. Of the patients with recurrent AAF (*n* = 14), 50% (7/14) undergo reablation, and two patients even twice. (B) Association of potential CIRs detected by the LP algorithm during the index tachycardia in recurrent tachycardias during the index procedure. In total, 13/33 patients showed a second flutter form; two patients develop even a third AAF form during the index procedure. In one case, the CIR is not detected by the LP algorithm. (C) Association of potential CIRs detected by LP algorithm during the index procedure and involvement in recurrent tachycardias during FU. In all de novo AAFs, the potential CIR was already detected by LP during the index procedure. AAF, atypical atrial flutter; AFib: atrial fibrillation; AT, atrial tachycardia; CIR, critical isthmus region; FU, follow‐up; LP, Lumipoint®.

### 3.4 Prediction of other ATs

Furthermore, we evaluated if suggested CIRs during the index procedure were involved in future clinical AAFs/ATs. As mentioned above, 60.6% (20/33) of patients had a recurrence of an AAF during the FU period of 10 ± 7 months (Figure 3A). In total 30.3% (*n* = 10) of the patients had a recurrence of AAF, 18.2% (*n* = 6) a recurrence of AFib, and 12.1% (*n* = 4) recurrence of both, AAF plus AFib. Of the patients with recurrent AAF (*n* = 14), 50% (7/14) underwent reablation (Figure 3A). Reasons for no reablation in FU (7/14), were the decision for AAD treatment (*n* = 2 flecainide; *n* = 1 amiodarone), the choice of no further treatment (*n* = 3). One patient underwent lung transplantation with several complications and so the patient was not really eligible for another ablation during FU.

Figure 3B illustrates the results of the association of potential CIRs detected by the LP algorithm during the index procedure and their involvement in subsequent AAF. During the index procedure, 12/33 patients developed a second flutter form and two patients showed even a third AAF form. In all but only one case, a relevant CIR was detected by the LP algorithm during the mapping of the first clinical AAF form.

Figure 3C illustrates the association of potential CIRs detected during the index procedure and their involvement in recurrent de novo AAF occurring during FU. In total *n* = 9 recurrent AAF forms in seven patients were examined, 4/9 were AAFs were recurrences of the index tachycardia, while 5/9 were new AAF forms. In all cases (5/5) of a novel recurrent AAF a corresponding matching CIR was already identified by the LP algorithm during the index procedure (Figure 3C).

## 4 DISCUSSION

Over the last few years, important advantages could be achieved by developing new high‐density mapping systems improving the treatment options for cardiac arrhythmias.^14^, ^15^ Mapping, facilitated by the rapid acquisition of thousands of electroanatomic points including activation time, allows the operator a deeper understanding of the arrhythmogenic mechanisms and pathogenesis. Taking the fact that ablation emerged as one of the most frequently used antiarrhythmic therapies, postablation tachycardias, mainly comprising complex re‐entry tachycardias, became more frequent.^16^, ^17^ Based on this, there is a clear demand for high‐density mapping features to facilitate precise ablation strategies. In this retrospective study, we evaluated the PALO and POR of a recently developed 3D LAT mapping‐based algorithm for the characterization and ablation guidance of complex ATs. We observed that the LP algorithm provides a promising tool for reliably detecting the CIR of a re‐entrant AAF and might carry the potential for the identification of arrhythmogenic substrate regions.

### 4.1 Patient and AAF characteristics

Consistent with the results of previous studies, a history of preablation or cardiac surgery was present in most of the studied patients.^5^, ^18^, ^19^, ^20^ Mean age in our study cohort was 71 years, which is rather old compared to patient cohorts of previous studies.^9^, ^15^, ^21^ In our study, 1.7 ATs per patient were detected and mapped. This is in line with previous studies, in which 1.3–1.6 AT forms per patient were reported.^9^, ^15^, ^21^, ^22^ The mean CL of our macro re‐entrant AT was 291 ms, which is also comparable to previous studies (CL range from 275 to 299 ms).^15^, ^21^

### 4.2 LP as a predictor of critical isthmus ablation regions

The “skyline” tool of LP produces a global activation histogram, which shows areas being activated at any given timeframe. Highlighted areas in the activation map, based on the minima of the global activation histogram are strongly related to slow conduction, lines of a block, or wavefront collision. In our study, 60.9% of all isthmi of all analyzed minima were located within the AAF re‐entry circuit. These results go along with data from previously published reports, in which 60%–67% of the isthmi were located in the AT re‐entry.^9^, ^21^

### 4.3 Role of electrical activation minima

To date, there are few published studies that investigated the LP “skyline” tool in patients with ATs.^9^, ^21^, ^22^ Regarding the definition of a minimum in previous studies, it can be stated, that the definitions, including ours, varied a lot. In one study minima were classified as valleys with EA ≤ 50% regardless of further characteristics.^21^ In another study, there was no precise definition of minima described.^9^

However, the number of minima reported in the previous studies per macro re‐entrant ATs was consistent with our observations. In our population, 2.4 minima per AT were detected, which is comparable to the ratio of 2 minima per ATs described in two other studies.^9^, ^21^ One of the previous studies did further characterize the electrical activation minima with a median minimum EA level of 20% and median width of 31 ms, which is quite consistent with our results (median minimum level 22% and median width 24.5 ms).^21^ The described number of isthmi per minimum differed between 1.4 and 1.6 in the other studies and so was also comparable to our result of 1.8 isthmi per minimum.^9^, ^22^ In our study, the PALO—suggested isthmi involves the CI—was very good with 98.2% with corresponds to the PALO of 100% in previous studies.^9^, ^21^ In contrast, the POR, stating that the LP algorithm shows only the CIR, was 12.3% quite low.

We analyzed different characteristics of our minima and tried to apply different preselection criteria to improve the POR. Selecting the broadest minima (35.9 ± 29.4 ms) resulted in a better POR of 49.1% but came along with decreased PALO down to 80.7%. By the selection of the smallest minimum (average 14.4 ± 9.8% EA), we were able to achieve an equivalent improved POR of 49.1% while still maintaining a quite good PALO of 91.2%.

Also, previous authors tried to further improve the quality of the proposed isthmi by preselection of the corresponding minimum. They showed that a smaller minimum was associated with decreased isthmus dimensions, conduction velocity, and increased EGM duration (*p* < 0.001). Even more, they showed that the level of minimum correlates closely with isthmus width, which is one of the most important CI characteristics.^21^ Another study demonstrated that setting a low threshold for the minimum (they suggested minima ≤20% of maximum EA) generates a better correlation with potential critical ablation sites. In macro re‐entry tachycardia, the POR was 62.8% if the minimum was ≤20% but only 15.0% if the minimum was >20%.^9^ These results are in line with our observations, taking the absolute minima of each AAF, characterized by ≤20% of maximum EA (in 75.4% of cases), we found a PALO of 95.3% and a POR of 60.5%. In postop patients, our LP algorithm showed even a slightly better, but not significant result with PALO 100% and POR 76.9%, when analyzing only the smallest minimum characterized by EA ≤ 20% (applicable in 13/14 AAF forms). A possible explanation might be that postop scars are more prominent and show a clear EA profile with scar‐related minima, which could lead to an advantage for our LP algorithm in postop patients when applying this parameter for the identification of the CIR.

Analyzing the broadest width (>50 ms) of each AAF, showed an excellent PALO as well as POR (100% and 90.0%, respectively), but a width of least 50 ms applied to only 17.5% of cases. Taken together selection of the smallest minima, represented by ≤20% of total EA, was commonly found and showed the best correlation with the CIR for a successful ablation leading to termination of the tachycardia.

### 4.4 LP as a predictor of future AAF

During the FU of 10 ± 7 months 42.3% of studied patients experienced a recurrence of an AAF, which is in line with previous data.^23^ Taken the high recurrence rates despite the high initial ablation success, it can be hypothesized that either initial ablation was not sufficient to create a complete isolation line or that initial bystander isthmus sites might become relevant in the future. To address this tissue, we evaluated if suggested isthmus sites during the index procedure were predictive of potential CIR of future clinically relevant ATs. Our analysis revealed that all recurrent de novo ATs (*n* = 5), which were reinvestigated and remapped in a second procedure, could be related to potential CIR areas already suggested by the LP algorithm during the index procedure. Nevertheless, taking the fact that by far not every isthmus detected during the initial procedure study (*n* = 248) was related to a clinically relevant AAF, the need for better understanding and preselection is given. Previous studies did not provide any data on this topic, therefore future studies should aim to evaluate the role of LP analysis with respect to the identification of arrhythmogenic substrates providing the pathophysiological basis for future arrhythmias.

### 4.5 Potential clinical implications

The occurrence of complex AT is frequent due to multiple iatrogenic scars, which lead to multiple conduction abnormalities that can be located within and outside the circuit as shown above.^9^ Conservative strategies are often frustrating and so underline the importance of interventional treatment.^5^ In scar areas, long‐duration EGMs are usually highly fragmented and so catching up with a meaningful time annotation can be challenging.^24^

LP algorithm was developed to facilitate the 3D course of a circulating wavefront in an easily interpretable 2D format.^21^ With a PALO of 98%, the preselection of potential ablation sites can help to identify a critical ablation site and thereby reduce mapping and procedure time. It could be helpful for less experienced operators to provide a guide. Based on our data, the LP algorithms can be used for pre‐selection, especially by applying additional criteria like depth and width to detect AE minima. Nevertheless, this has still to be combined with the physician's interpretation of the re‐entry and the potentially most promising ablation area for persistent and fast success. In addition, our preliminary results suggest that LP might also be a tool for substrate characterization, which might become relevant for future arrhythmia development; however, this is a matter of future research.

### 4.6 Limitations

This retrospective study is derived from a single‐center experience and involves a limited number of patients and AAFs. Limitations apply for dual loop tachycardias, in which individual wavefronts can be maximally synchronized when they pass through a shared isthmus and so then overall EA is often minimal but does not necessarily represent the perfect ablation site. Furthermore, wavefront collisions, isthmi outside the re‐entry tachycardia, and undersampling can be reasons for misleading minima.^21^ Randomized studies are needed to evaluate the benefit in facilitate ablation procedures and improving outcomes. Especially if ablation of additional isthmi during index procedure might lead to a reduction of recurrence of other AT forms in the future has to be evaluated in further studies.

## 5 CONCLUSION

The novel algorithm LP developed for the Rhythmia® mapping system provides new insights into the characterization and illustration of CIRs of complex ATs. In our study, the tool showed an excellent PALO (98.2%), but poor overall POR (12.3%) to detect CIR of the macro re‐entry tachycardia. However, POR could be significantly improved up to 60% accompanied by a still quite good PALO of 95% when selecting only the lowest EA minimum characterized by EA ≤ 20%.

In addition, initially detected irrelevant bystander CIRs during the first AAF procedure might become relevant for future atrial flutter forms potentially identifying an arrhythmogenic substrate. Identification of target areas in the activation map bears the potential to provide a good preselection for potential critical isthmus regions improving procedural parameters and ablation success.

## CONFLICT OF INTEREST STATEMENT

Reza Wakili has received consultant fees, speaking honoraria, and travel expenses from Biotronik; Boston Scientific and Medtronic; investigator‐initiated funding for research projects (initiated by him) from Bristol‐Myers Squibb/Pfizer and Boston Scientific. The remaining authors declare no conflict of interest.

## Supporting information

Supplementary information.

Supplementary information.

## Data Availability

The data that support the findings of this study are available from the corresponding author upon reasonable request.

## References

[1] Chugh A , Oral H , Lemola K , et al. Prevalence, mechanism, and clinical significance of macroreentrant atrial tachycardia during and following left trial ablation for atrial fibrillation. Heart Rhythm. 2005;2:464‐471. 15840468 10.1016/j.hrthm.2005.01.027

[2] Patel AM , d'Avila A , Neuzil P , et al. Atrial tachycardia after ablation of persistent atrial fibrillation. Circ Arrhythmia Electrophysiol. 2008;1(1):14‐22. 10.1161/CIRCEP.107.748160 19808389

[3] Jaïs P , Matsuo S , Knecht S , et al. A deductive mapping strategy for atrial tachycardia following atrial fibrillation ablation: importance of localized re‐entry. J Cardiovasc Electrophysiol. 2009;20(5):480‐491. 19207747 10.1111/j.1540-8167.2008.01373.x

[4] Saghy L , Tutuianu C , Szilagyi J . Atrial tachycardias following atrial fibrillation ablation. Curr Cardiol Rev. 2014;11(2):149‐156. 10.2174/1573403X10666141013122400 PMC4356722 25308808

[5] Rostock T , Drewitz I , Steven D , et al. Characterization, mapping, and catheter ablation of recurrent atrial tachycardias after stepwise ablation of long‐lasting persistent atrial fibrillation. Circ Arrhythmia Electrophysiol. 2010;3(2):160‐169. 10.1161/CIRCEP.109.899021 20133933

[6] Wakili R , Voigt N , Kääb S , Dobrev D , Nattel S . Recent advances in the molecular pathophysiology of atrial fibrillation. J Clin Invest. 2011;121(8):2955‐2968. 21804195 10.1172/JCI46315 PMC3148739

[7] Shah D . Twelve‐lead ECG interpretation in a patient with presumed left atrial flutter following AF ablation. J Cardiovasc Electrophysiol. 2011;22(5):613‐617. 21235664 10.1111/j.1540-8167.2010.01982.x

[8] Martin CA , Takigawa M , Martin R , et al. Use of novel electrogram “Lumipoint” algorithm to detect critical isthmus and abnormal potentials for ablation in ventricular tachycardia. JACC Clin Electrophysiol. 2019;5:470‐479. 31000101 10.1016/j.jacep.2019.01.016

[9] Takigawa M , Martin CA , Derval N , et al. Insights from atrial surface activation throughout atrial tachycardia cycle length: a new mapping tool. Heart Rhythm. 2019;16:1652‐1660. 31004777 10.1016/j.hrthm.2019.04.029

[10] Hocini M , Jaïs P , Sanders P , et al. Techniques, evaluation, and consequences of linear block at the left atrial roof in paroxysmal atrial fibrillation: a prospective randomized study. Circulation. 2005;112(24):3688‐3696. 16344401 10.1161/CIRCULATIONAHA.105.541052

[11] Jaïs P , Hocini M , Hsu LF , et al. Technique and results of linear ablation at the mitral isthmus. Circulation. 2004;110(19):2996‐3002. 15520313 10.1161/01.CIR.0000146917.75041.58

[12] Jaïs P , Hocini M , O'Neill MD , et al. How to perform linear lesions. Heart Rhythm. 2007;4(6):803‐809. 17556210 10.1016/j.hrthm.2007.01.021

[13] Laţcu DG , Bun SS , Viera F , et al. Selection of critical isthmus in Scar‐Related atrial tachycardia using a new automated ultrahigh resolution mapping system. Circ Arrhythmia Electrophysiol. 2017;10(1):e004510. 10.1161/CIRCEP.116.004510 28039280

[14] Meyer C . High‐density mapping‐based ablation strategies of cardiac rhythm disorders: the RHYTHMIA™ experience at new horizons. EP Europace. 2019;21(Suppl_3):iii7‐iii10. 10.1093/europace/euz154 31400216

[15] Schaeffer B , Hoffmann BA , Meyer C , et al. Characterization, mapping, and ablation of complex atrial tachycardia: initial experience with a novel method of ultra high‐density 3D mapping. J Cardiovasc Electrophysiol. 2016;27(10):1139‐1150. 27325527 10.1111/jce.13035

[16] Takigawa M , Derval N , Frontera A , et al. Revisiting anatomic macroreentrant tachycardia after atrial fibrillation ablation using ultrahigh‐resolution mapping: implications for ablation. Heart Rhythm. 2018;15(3):326‐333. 29081399 10.1016/j.hrthm.2017.10.029

[17] Takigawa M , Derval N , Maury P , et al. Comprehensive multicenter study of the common isthmus in post‐atrial fibrillation ablation multiple‐loop atrial tachycardia. Circ Arrhythmia Electrophysiol. 2018;11(6):e006019. 10.1161/CIRCEP.117.006019 29769223

[18] Rostock T , Steven D , Hoffmann B , et al. Chronic atrial fibrillation is a biatrial arrhythmia: data from catheter ablation of chronic atrial fibrillation aiming arrhythmia termination using a sequential ablation approach. Circ Arrhythmia Electrophysiol. 2008;1(5):344‐353. 10.1161/CIRCEP.108.772392 19808429

[19] Mesas CéE , Pappone C , Lang CCE , et al. Left atrial tachycardia after circumferential pulmonary vein ablation for atrial fibrillation. J Am Coll Cardiol. 2004;44(5):1071‐1079. 15337221 10.1016/j.jacc.2004.05.072

[20] Haissaguerre M , Hocini M , Sanders P , et al. Catheter ablation of long‐lasting persistent atrial fibrillation: clinical outcome and mechanisms of subsequent arrhythmias. J Cardiovasc Electrophysiol. 2005;16(11):1138‐1147. 16302893 10.1111/j.1540-8167.2005.00308.x

[21] Moore JP , Buch E , Gallotti RG , Shannon KM . Ultrahigh‐density mapping supplemented with global chamber activation identifies noncavotricuspid‐dependent intra‐atrial re‐entry conduction isthmuses in adult congenital heart disease. J Cardiovasc Electrophysiol. 2019;30(12):2797‐2805. 31646694 10.1111/jce.14251

[22] Alken FA , Klatt N , Muenkler P , et al. Advanced mapping strategies for ablation therapy in adults with congenital heart disease. Cardiovasc Diagn Ther. 2019;9(Suppl 2):S247‐S263. 31737533 10.21037/cdt.2019.10.02 PMC6837939

[23] Sultan A , Lüker J , Andresen D , et al. Predictors of atrial fibrillation recurrence after catheter ablation: data from the German Ablation Registry. Sci Rep. 2017;7(1):16678. 29192223 10.1038/s41598-017-16938-6 PMC5709464

[24] Bisceglia C , Frontera A , Della Bella P . The LUMIPOINT™ software: are we just at the turning point? EP Europace. 2019;21(Suppl_3):iii25‐iii26. 10.1093/europace/euz144 31400215

