# Supplementary material for: Characterization of temporal electrical activity patterns for detection of critical isthmus regions of recurrent atypical atrial flutter
Source: Clin Cardiol. 2023 Mar 27;46(5):574–83. doi: 10.1002/clc.24009 (PMC10189070; doi:10.1002/clc.24009)
Supplement: Supplementary file 1 — Supplementary information. [file CLC-46-574-s001.pdf]

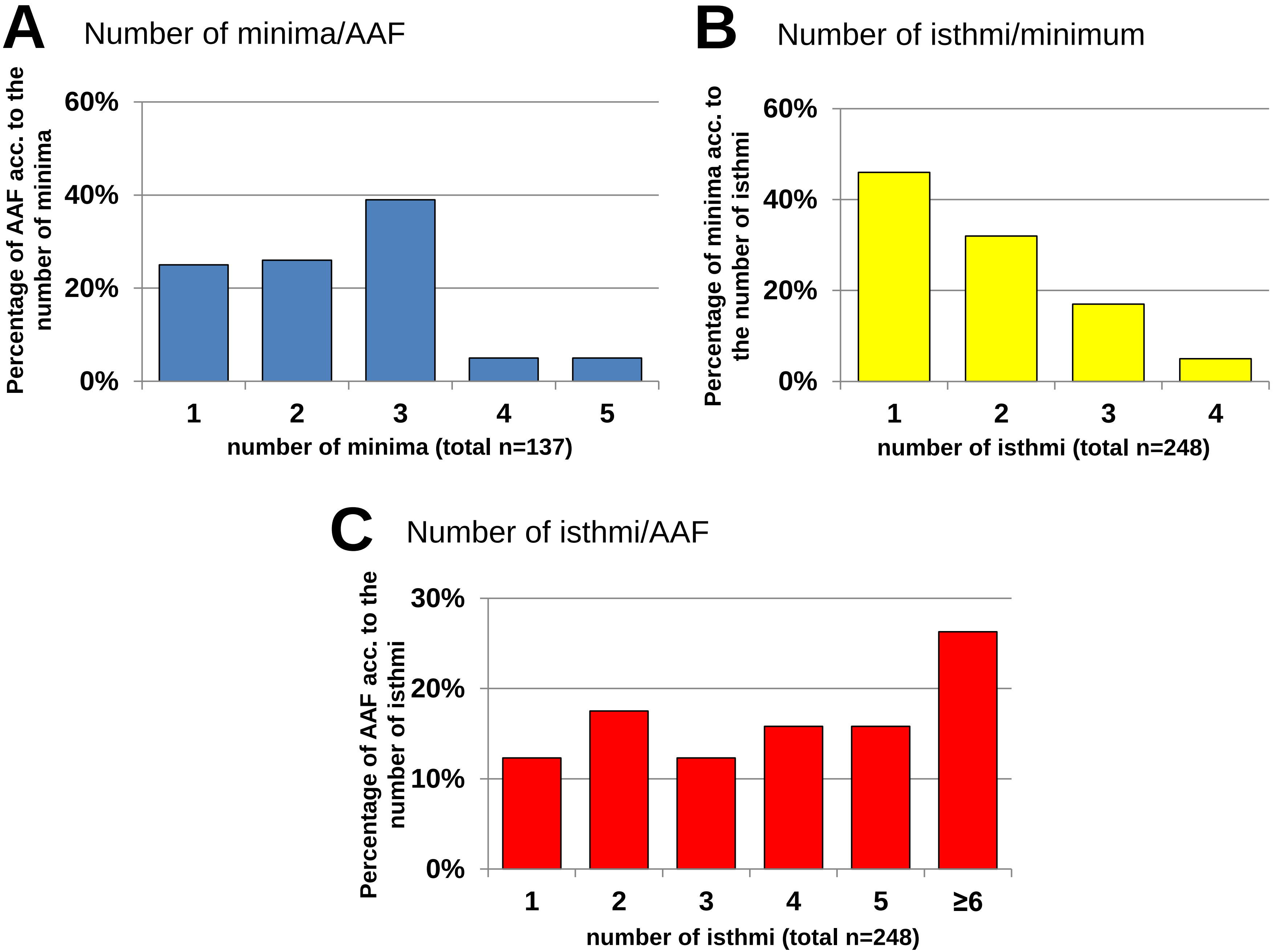

Supplementary figure 1

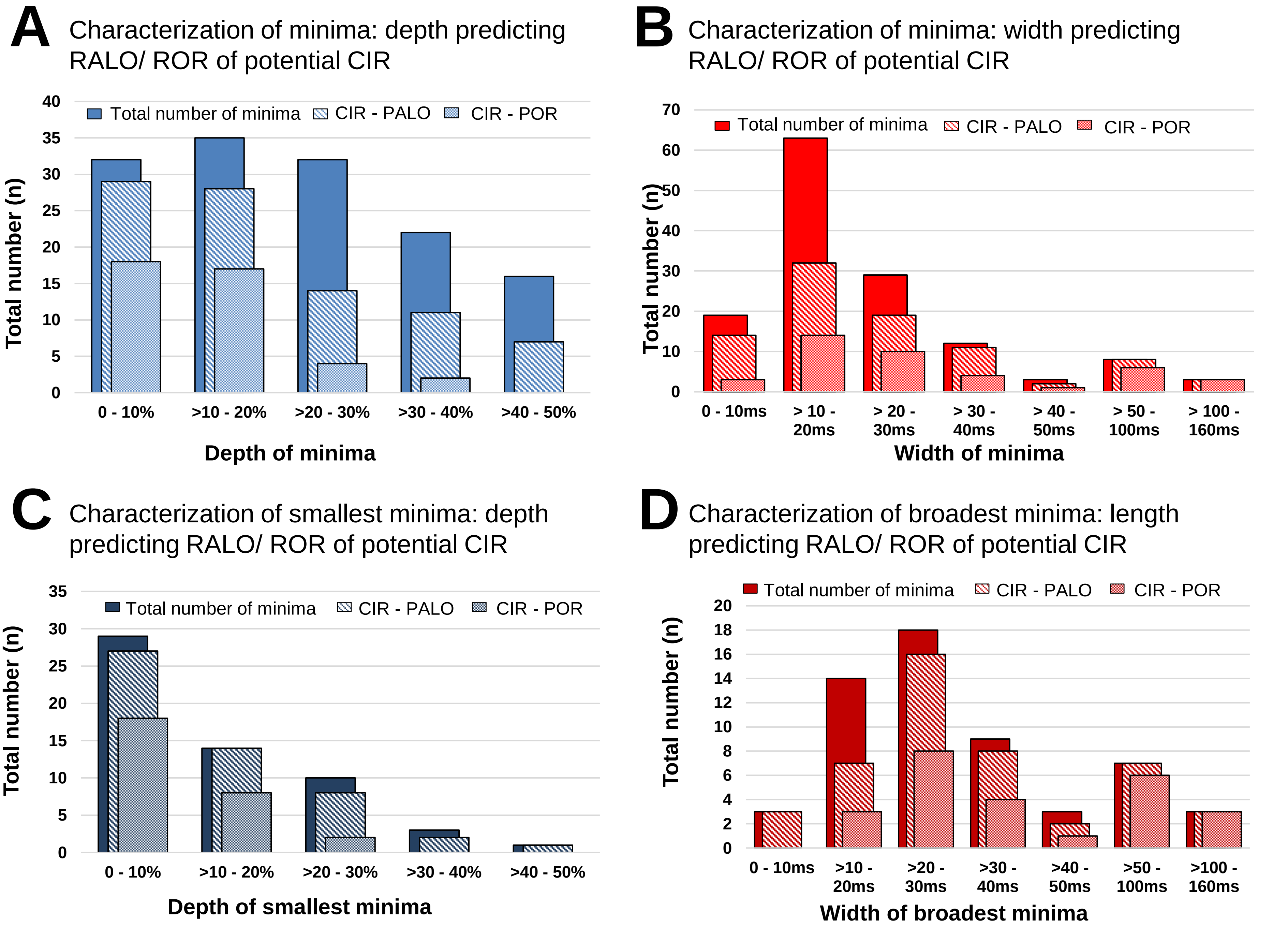

Supplementary figure 2

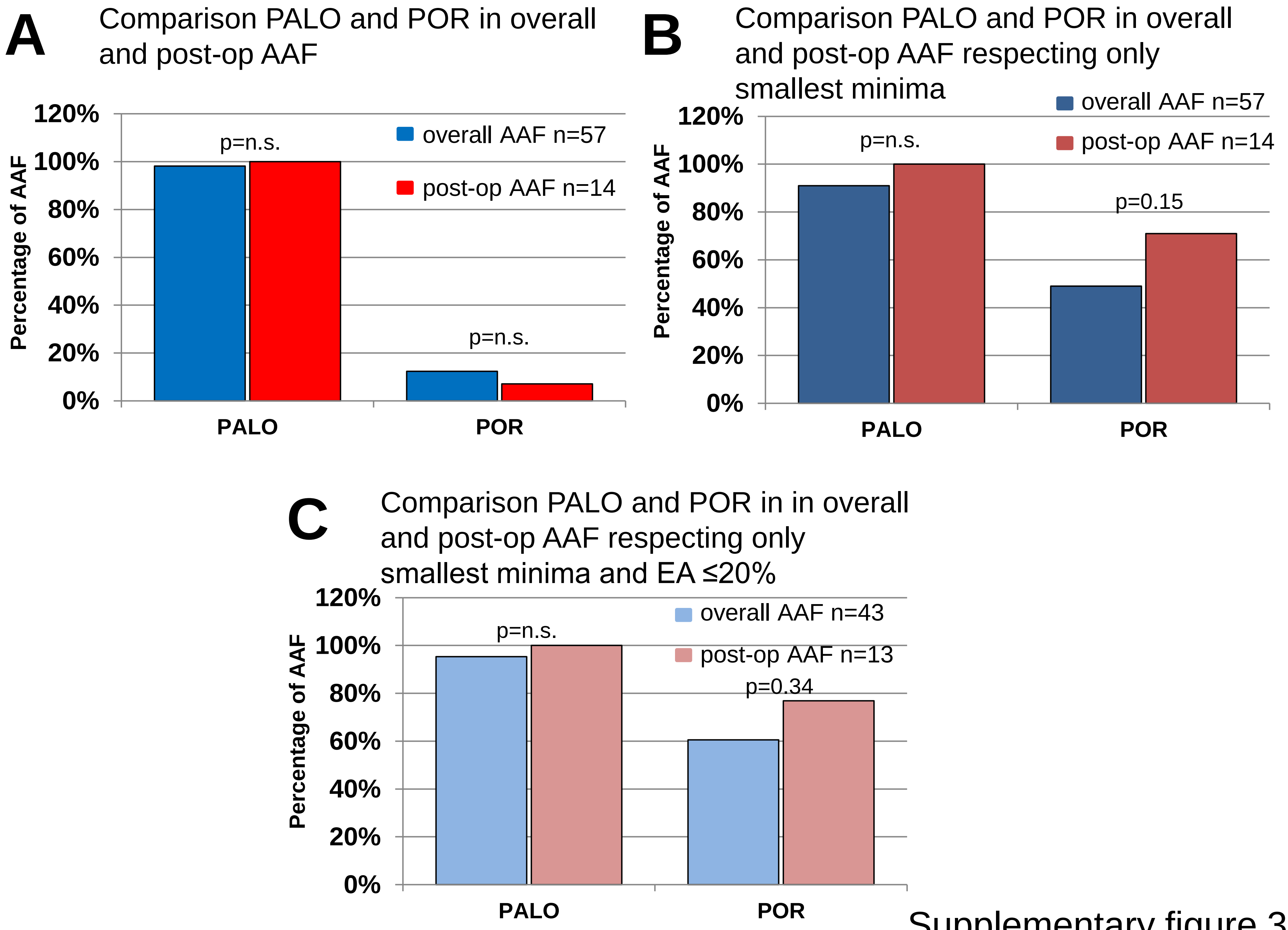

Supplementary figure 3
