# Supplementary material for: Characterization of temporal electrical activity patterns for detection of critical isthmus regions of recurrent atypical atrial flutter
Source: Clin Cardiol. 2023 Mar 27;46(5):574–83. doi: 10.1002/clc.24009 (PMC10189070; doi:10.1002/clc.24009)
Supplement: Supplementary file 2 — Supplementary information. [file CLC-46-574-s002.pdf]

Legends supplementary Figures:

Supplementary figure 1: Distribution of minima and isthmi. (A) The skylines involve a total number of minima of 137. In most cases, 3 minima per AAF (39%) or 2 minima/AAF (26%). (B) The total number of highlighted areas corresponding to potential isthmi was 248. Analyses of the distribution show that in most cases 1 isthmus/minimum was shown (46%). (C) shows isthmi per AAF by decreasing order, most commonly the number is: ≥6 isthmi/AAF (26%). AAF: atypical atrial flutter.

*Supplementary figure 2: Characterization of minima predicting the potential CIR. (A) Detailed analysis regarding the role of depth of the minima; deepest minimum with higher POR as well as PALO. (B) Detailed analysis regarding role of length; longer minima are associated with a higher PALO as well as POR in contrast to shorter minima. (C) Detailed analysis regarding role of deepest minima of each AAF; deeper minima result in a better PALO and POR in comparison to higher minima. (D) Detailed analysis regarding role of width of minima; longer minima can better predict potential CIR than shorter minima. CIR: critical isthmus region; PALO: **p**robability of identifying **a**t **l**east **o**ne CIR; POR: **p**robability of identifying **o**nly the **r**elevant CIR; AAF: atypical atrial flutter.*

*Supplementary figure 3: Comparison PALO and POR between AFF forms in patients after cardiac surgery and overall AAF forms: 8 patients (24.2%) were post-cardiac surgery and developed 14 AAF forms. (A) comparison for PALO and POR in overall AAF forms (PALO 98.2% overall vs. 100% post-op, p=1.0; POR 12.3% overall and 7.1% post-op, p=1.0). (B), analysis of smallest minimum in the two subgroups post-op AAF vs. AAF forms (PALO 91.2% overall vs. 100% post-op, p=0.57; POR 49.1% overall vs. 71.4% post-op; p=0.15). (C) analysis for minima characterized by EA≤20% in post-op AAF (n=13) compared to overall AAF (n=43) (PALO 95.3% overall vs. 100% post-op, p=1.0; POR 60.5% overall vs. 76.9% post-op, p=0.34). AAF: atypical atrial flutter; EA: electrical activity; PALO: **p**robability of identifying **a**t **l**east **o**ne CIR; POR: **p**robability of identifying **o**nly the **r**elevant CIR.*
